# Supplementary material for: The role of ATP-binding Cassette subfamily B member 6 in the inner ear
Source: Nat Commun. 2024 Nov 18;15:9885. doi: 10.1038/s41467-024-53663-x (PMC11574312; doi:10.1038/s41467-024-53663-x)
Supplement: Supplementary file 1 — Supplementary Information [file 41467_2024_53663_MOESM1_ESM.pdf]

# Supplementary Information

## The role of ATP-binding Cassette subfamily B member 6 in the inner ear

Stefanie A. Baril[1], Katie A. Wilson[2,3], Md Munan Shaik[4], Yu Fukuda[1], Robyn A. Umans[5], Alessandro Barbieri[6,7], John Lynch[1], Tomoka Gose[1], Alexander Myasnikov[4], Michael L. Oldham[4], Yao Wang[1], Jingwen Zhu[1,8], Jie Fang,[9,10] Jian Zuo[9], Ravi C. Kalathur[4], Robert C. Ford[6], Allison Coffin[11], Michael R. Taylor[5,12], Megan L. O'Mara[2,13], John D. Schuetz[1*]

[1]Department of Pharmaceutical Sciences, St. Jude Children's Research Hospital, Memphis, TN, USA
[2]Research School of Chemistry, College of Science, The Australian National University, Canberra, Australian Capital Territory, Australia
[3]Department of Biochemistry, Memorial University of Newfoundland, St. John's, Newfoundland and Labrador, Canada.
[4]Department of Structural Biology, St. Jude Children's Research Hospital, Memphis, TN, USA
[5]Department of Chemical Biology and Therapeutics, St. Jude Children's Research Hospital, Memphis, TN, USA
[6]School of Biological Sciences, Faculty of Biology Medicine and Health, The University of Manchester, Manchester, UK
[7]Bioinformatics Institute (BII), Agency for Science, Technology, and Research (A*STAR), Singapore
[8]Department of Pharmaceutical Sciences, University of Tennessee Health Science Center, Memphis, TN, USA.
[9]Department of Developmental Neurobiology, St. Jude Children's Research Hospital, Memphis, TN, USA
[10]Department of Surgery, St. Jude Children's Research Hospital, Memphis, TN, USA
[11]Department of Integrative Physiology and Neuroscience, Washington State University Vancouver, Vancouver, WA, USA
[12]School of Pharmacy, Pharmaceutical Sciences Division, University of Wisconsin-Madison, Madison, WI, USA
[13]Australian Institute for Bioengineering and Nanotechnology, The University of Queensland, St. Lucia, Queensland, Australia

# Table of Contents

**Supplementary Methods** .......... 2
**Supplementary Table 1.** Predicted effects of DUH mutations on ABCB6 function .......... 4
**Supplementary Table 2.** Cryo-EM data collection, refinement, and validation statistics .......... 5
**Supplementary Table 3.** Residues that are in contact with L356 during the MD simulations of ABCB6 .......... 6
**Supplementary Table 4.** Phenotypes of zebrafish Abcb6 morphants from Fig. 5c .......... 6
**Supplementary Table 5.** PCR primers for DUH mutations .......... 7
**Supplementary Table 6.** Treatment conditions to stabilize L356P .......... 7
**Supplementary Table 7.** Composition of each of the systems simulated .......... 8
**Supplementary Table 8.** Phenotypes of zebrafish Abcb6 morphants from Supplementary Fig. 12c .......... 8
**Supplementary Figure 1.** Purification of ABCB6 .......... 9
**Supplementary Figure 2.** Cryo-EM image processing .......... 10
**Supplementary Figure 3.** Cryo-EM refinement .......... 11
**Supplementary Figure 4.** Cryo-EM reconstruction .......... 12
**Supplementary Figure 5.** Additional analysis of the L356P mutation .......... 13
**Supplementary Figure 6.** Additional characterization of zebrafish Abcb6 .......... 14
**Supplementary Figure 7.** Analysis of zebrafish Abcb6 homology model. .......... 15
**Supplementary Figure 8.** Comparison of zebrafish homology model and AlphaFold model .......... 15
**Supplementary Figure 9.** RT-PCR analysis of morpholino oligonucleotides .......... 16
**Supplementary Figure 10.** Analysis of zebrafish hair cell numbers .......... 17
**Supplementary Figure 11.** Morpholino knockdown reduced lateral line cell number .......... 18
**Supplementary Figure 12.** 2-saccule phenotype is dependent on MO15 dose, but not MO7 dose .......... 19
**Supplementary Figure 13.** Expression of heme synthesis genes increases with development in the cochlea .......... 20
**Supplementary Figure 14.** RNA-seq shows several hearing-related genes are affected by KO of Abcb6 .......... 21
**Supplementary Figure 15.** Normalized mRNA read counts from various members of the ABC B-subfamily .......... 22
**Supplementary Figure 16.** *o*-Dianisidine staining does not reflect a change in red blood cells .......... 23
**Supplementary Figure 17.** Time-dependent RMSD of human and zebrafish ABCB6 simulations .......... 24
**Supplementary References** .......... 25

## Supplementary Methods

### Calculations with ATP-agarose and Hemin-agarose Data

Signal of the input and eluted bands were quantitated using ImageStudio Software V5.2.5. Percent of WT Binding was calculated as follows:

$$\text{\% of WT binding} = \left[\left(\frac{\text{Mutant eluted signal}}{\text{Mutant input signal}}\right) \div \left(\frac{\text{WT eluted signal}}{\text{WT input signal}}\right)\right] \times 100\%$$

### Calculations with Proteasomal and Lysosomal Inhibitors

Signal of the input and eluted bands were quantitated using ImageStudio Software V5.2.5. Percent of untreated signal was calculated as follows for each protein (ABCB6 WT and L356P):

$$\text{\% of untreated signal} = \left[\left(\frac{\text{Treatment ABCB6 signal}}{\text{Treatment GAPDH signal}}\right) \div \left(\frac{\text{Untreated ABCB6 signal}}{\text{Untreated GAPDH signal}}\right)\right] \times 100\%$$

### Live Cell Imaging

MEF *Abcb6* KO cells were seeded in ibidi 4-well chambers. The next day, 180 ng pcDNA-3.1-zfAbcb6-GFP plasmid[1] was transfected by Fugene reagent according to manufacturer's instructions. 20 hours after transfection, WGA (final conc 8 µg/ml) was added and incubated for 5 minutes at 37°C to label the plasma membrane. Live cell imaging was then performed with confocal microscopy.

### Assessing Morpholino Knockdown Efficacy by RT-PCR

The knockdown effectiveness of the *abcb6a* splice donor MOs was assessed by reverse transcription polymerase chain reaction (RT-PCR). Following microinjection, control and morpholino injected embryos at 3 days post fertilization (dpf) were anesthetized in 0.02% Tricaine, transferred into RNase/DNase-free 1.5 ml microcentrifuge tubes with fitted pestle (Fisher Scientific, K749521-1500), homogenized in TRIzol, and total RNA was extracted according to the manufacturer's protocol (Invitrogen, 15596026). cDNA was synthesized by reverse transcription using the SuperScript IV First-Strand Synthesis System using Oligo(dT) primers according to the manufacturer's protocol (Invitrogen, 18091050). To examine altered splicing by the MOs, the following DNA primers were designed using Primer3[2] to PCR amplify *abcb6a* cDNA across the targeted splice donor sites: ex7/in7 forward primer 5'-TCGCTCATCTGCATGCTCTA-3', ex7/in7 reverse primer 5'-AGCTTTGGATTTGGCGTTGT-3', ex15/in15 forward primer 5'-TTCAAGCTCTTCACCGAGGA-3', and ex15/in15 reverse primer 5'-TTCTCCTCCGCTTAGCTTCA-3'. The following primers designed against *actin b1* (*actb1*) were used as a control: *actb1* forward primer 5'-CCCTCCATTGTTGGACGAC-3' and *actb1*reverse primer 5'-CCGATCCAGACGGAGTATTTG-3'. From wildtype cDNA,

the ex7/in7, ex15/in15, and *actb1* primers PCR amplify 311 bp, 501 bp, and 932 bp fragments, respectively.

**Staining and Quantitation of Hair Cells**

Fish were euthanized at 3 (dpf) and fixed overnight in 4% paraformaldehyde at 4°C. Fish were labeled with a rabbit polyclonal antibody to myosin 7a, a commonly used hair cell marker.[3-5] Briefly, fish were rinsed in phosphate-buffered saline (PBS), followed by a rinse in distilled water. Fish were blocked in PBS containing 10% goat serum and 0.1% triton, then incubated in anti-myosin7a (1:1000 dilution; Proteus Biosciences) in PBS with 1% goat serum and 0.1% triton; incubation was performed for 1 hour at 37 °C, then overnight at 4 °C. Fish were rinsed in PBS with 0.1% triton, then incubated in Alexa 488 goat anti-rabbit (1:500 dilution; Invitrogen) in PBS with 1% goat serum and 0.1% triton for 4 hours at room temperature. Fish were further rinsed and stored in 1:1 mixture of PBS glycerol. Lateral line hair cells were quantified as described in the main methods. Example neuromasts were imaged on a Leica SP8 laser scanning confocal microscope.

**Staining Erythroid Cells with *o*-Dianisidine**

The level of hemoglobin in erythroid cells was evaluated by *o*-dianisidine staining as previously described.[6] Briefly, live embryos were transferred to glass vials and stained in the dark for 30 min at room temperature in a solution 0.06% *o*-dianisidine, 0.01 M sodium acetate (pH 4.5), 0.6% $H_2O_2$, and 40% (vol/vol) ethanol. The embryos were washed three times in $dH_2O$, fixed in 4% PFA/PBS for 1 hour, rinsed three times in PBST, and then imaged on a Nikon SMZ18 stereomicroscope equipped with a Nikon DS-Fi2 color camera.

**Isolation of Cochlea for RNA-Seq**

Each mouse was euthanized with carbon dioxide. The mouse head was cut in half and the brain was removed from the skull. The skull was positioned face up and the junctions between the skull and the temporal bone were pushed away from the cochlear area to break the cochlea away from skull. Extra bone or tissue were then removed from the cochlea.

**Isolation of Cochlear RNA**

The cochlea was disrupted with a homogenizer in 500 µL of Qiagen Buffer RLT. 500 µL of 70% ethanol was added to the lysate and the solution was mixed via pipette. The sample (including any precipitate) was added to a RNeasy mini spin column placed in a 2 mL collection tube. The tube was centrifuged for 15 seconds at ≥8000 x *g*. The sample was then purified according to manufacturer's instructions on the Qiagen RNeasy Mini Kit, and the sample was eluted into 30 µL RNase-free water.

**RNA-Seq analysis**

Read counts per gene are analyzed using R software using the following methods: Only confidently annotated (level 1 (verified) and 2 (manually annotated) gene annotation), protein-coding genes are considered in the standard differential expression analysis. Low count genes were removed from analysis using a library.size adjusted cutoff. Normalization factors were generated using the TMM method. Counts were normalized using voom. Voom normalized counts were analyzed using the lmFit and eBayes functions of the limma software package. MA plots were generated from voom normalized data and volcano plots generated from voom normalized data plus p-values values from limma.

**Supplementary Table 1.** Predicted effects of DUH mutations on ABCB6 function. Predictions were generated using PolyPhen-2 (http://genetics.bwh.harvard.edu/pph2/), SIFT sequence (https://sift.bii.a-star.edu.sg/www/SIFT_seq_submit2.html), and Mutation Accessor (http://mutationassessor.org/r3/).[7-9]

| Mutation | PolyPhen-2 | SIFT | Mutation Accessor |
|---|---|---|---|
| S170G | Benign | Affect Function | Low Effect |
| S322R | Possibly Damaging | Tolerated | Medium Effect |
| L356P | Probably Damaging | Affect Function | High Effect |
| Y424H | Probably Damaging | Affect Function | High Effect |
| A453V | Probably Damaging | Tolerated | Medium Effect |
| Q555K | Probably Damaging | Tolerated | Low Effect |
| G579E | Benign | Tolerated | Low Effect |

**Supplementary Table 2.** Cryo-EM data collection, refinement, and validation statistics.

| | |
|---|---|
| **Data collection** | |
| Microscope | Titan Krios (FEI) |
| Voltage (kV) | 300 |
| Detector | K3 Summit (Gatan) |
| Pixel size (Å) | 1.087 |
| Defocus range (μm) | -1.5 to -2.5 |
| Movies | 6807 |
| Frames/movie | 60 |
| Dose rate (electrons/pix/s) | 30 |
| Total dose (electrons/Å$^2$) | 66 |
| **Model Composition** | |
| Chains | 2 |
| Non-hydrogen atoms | 10062 |
| Protein residues | 1182 |
| Lipids/detergents/ligands | 20 |
| **Refinement** | |
| Number of particles | 1,587,831 |
| Resolution (Å) | 2.93 |
| RMS deviations | |
| Bond length (Å) (# > 4σ) | 0.003 (0) |
| Bond angles (°) (# > 4σ) | 0.633 (0) |
| **Validation** | |
| MolProbity score | 1.54 |
| Clash score | 6.83 |
| Ramachandran plot (%) | |
| Outliers | 0.00 |
| Allowed | 2.89 |
| Favored | 97.11 |
| Rotamers (%) | |
| Outliers | 0.0 |
| Favored | 99.9 |
| Cβ outliers (%) | 0 |
| Peptide plane (%) | |
| Cis proline/general | 0/0 |
| Twisted proline/general | 0/0 |

**Supplementary Table 3**. Average number of hydrogen bonds that are formed involving the conserved regions of the ABCB6 NBD during the MD simulations of ABCB6. A reduction in hydrogen bonding is observed between the WT and L356P for the highly conserved residues in the Walker A, Walker B, and Q-loop, motifs.

| | WT ABCB6 | L356P Mutant |
|---|---|---|
| Walker A | 26 ± 4 | 23 ± 4 |
| Q-loop | 12 ± 4 | 10 ± 3 |
| ABC-Signature | 22 ± 4 | 22 ± 4 |
| Walker B | 24 ± 2 | 20 ± 2 |
| H-loop | 24 ± 4 | 28 ± 4 |

**Supplementary Table 4.** Phenotypes of zebrafish Abcb6 morphants from Fig. 5c.

| | % Fish with 2 Saccules + 0 Utricles | % Fish with 2 Saccules + 1 Utricle | % Fish with 2 Saccules + 2 Utricles |
|---|---|---|---|
| Uninjected Control | 0 | 0 | 100 |
| Zebrafish *abcb6* mRNA | 0 | 0 | 100 |
| Zebrafish *abcb6* Morpholino | 95.0 | 5.0 | 0 |
| Zebrafish *abcb6* Morpholino + human *ABCB6* mRNA | 5.3 | 42.1 | 52.6 |

**Supplementary Table 5.** PCR primers for DUH mutations. The single nucleotide polymorphism responsible for the DUH variant is shown as a red lower-case letter.

| Primer Name | Primer Sequence |
|---|---|
| huB6_S170G_F | GGAACgGCCCACAGTGGTG |
| huB6_S170G_R | GGGCcGTTCCAAGACACCAG |
| huB6_S322R_F | GGCcGTACAGGCTTCGTGAG |
| huB6_S322R_R | GTACgGCCAGTGCCACCC |
| huB6_L356P_F | GAGCcCTCACTGCGCTGG |
| huB6_L356P_R | GTGAGgGCTCGTGCAGGTG |
| huB6_Y424H_F | GTCTTcACCTCACCCTGACCATTGTG |
| huB6_Y424H_R | GAGGTgAAGACTCATGCACAGGAACAC |
| huB6_A453V_F | CACGAGtAGTGGACTCTCTGCTAAAC |
| huB6_A453V_R | CCACTaCTCGTGCCCGGGTAG |
| huB6_Q555K_F | GGATGATCaAGACCAACTTCATTGACATGGAGAACATG |
| huB6_Q555K_R | GGTCTtGATCATCCTGTAGTAGGTGCCAAAC |
| huB6_G579E_F | CTGaAGCAGGGCCCCTTCG |
| huB6_G579E_R | GCTtCAGGAAGGTCCTTCACTTCTGTCTC |

**Supplementary Table 6.** Treatment conditions to stabilize L356P. T=0 corresponds to time transfection occurred.

| Compound Name | Final Concentration | Time of Treatment * | Duration of Treatment |
|---|---|---|---|
| MG132 | 1 µM | 20 hr. | 4 hr. |
| 4-Phenylbutyrate (4-PBA) | 2 mM | 1 hr. | 23 hr. |
| Chloroquine (CQ) | 100 µM | 1 hr. | 23 hr. |

**Supplementary Table 7.** Composition of each of the systems simulated.

| | ABCB6 WT | ABCB6 L356P | Zebrafish Abcb6 WT | Zebrafish Abcb6 L356P |
|---|---|---|---|---|
| Number of Abcb6 | 1 | 1 | 1 | 1 |
| Number of CHOL Lipids | 165 | 180 | 169 | 174 |
| Number of OAPE Lipids | 265 | 290 | 273 | 281 |
| Number of PSPC Lipids | 238 | 260 | 246 | 252 |
| Salt Concentration | 0.15 M | 0.15 M | 0.15 M | 0.15 M |
| Number of Na+ ions | 171 | 191 | 185 | 192 |
| Number of Cl- ions | 179 | 199 | 185 | 192 |
| Number of Water Molecules | 66656 | 73888 | 68825 | 71476 |
| Total number of atoms | 298125 | 327178 | 306810 | 317083 |
| Simulation box dimensions | 15 Å x 14 Å x 17 Å | 16 Å x 15 Å x 17 Å | 15 Å x 15 Å x 16 Å | 15 Å x 15 Å x 17 Å |

**Supplementary Table 8.** Phenotypes of zebrafish Abcb6 morphants from Supplementary Figure 12c.

| | Total Fish | Fish with 2 Saccules + 2 Utricles | Fish with 2 Saccules + 0 Utricles | % Fish with 2 Saccules + 2 Utricles | % Fish with 2 Saccules + 0 Utricles |
|---|---|---|---|---|---|
| Uninjected Control | 320 | 320 | 0 | 100% | 0% |
| Control MO | 132 | 132 | 0 | 100% | 0% |
| MO15 8 ng | 74 | 0 | 74 | 0% | 100% |
| MO15 4 ng | 191 | 2 | 189 | 1% | 99% |
| MO15 2 ng | 177 | 10 | 167 | 6% | 94% |
| MO15 1 ng | 127 | 67 | 60 | 53% | 47% |
| MO15 0.5 ng | 113 | 104 | 9 | 92% | 8% |
| MO7 8 ng | 66 | 66 | 0 | 100% | 0% |
| MO7 4 ng | 131 | 131 | 0 | 100% | 0% |

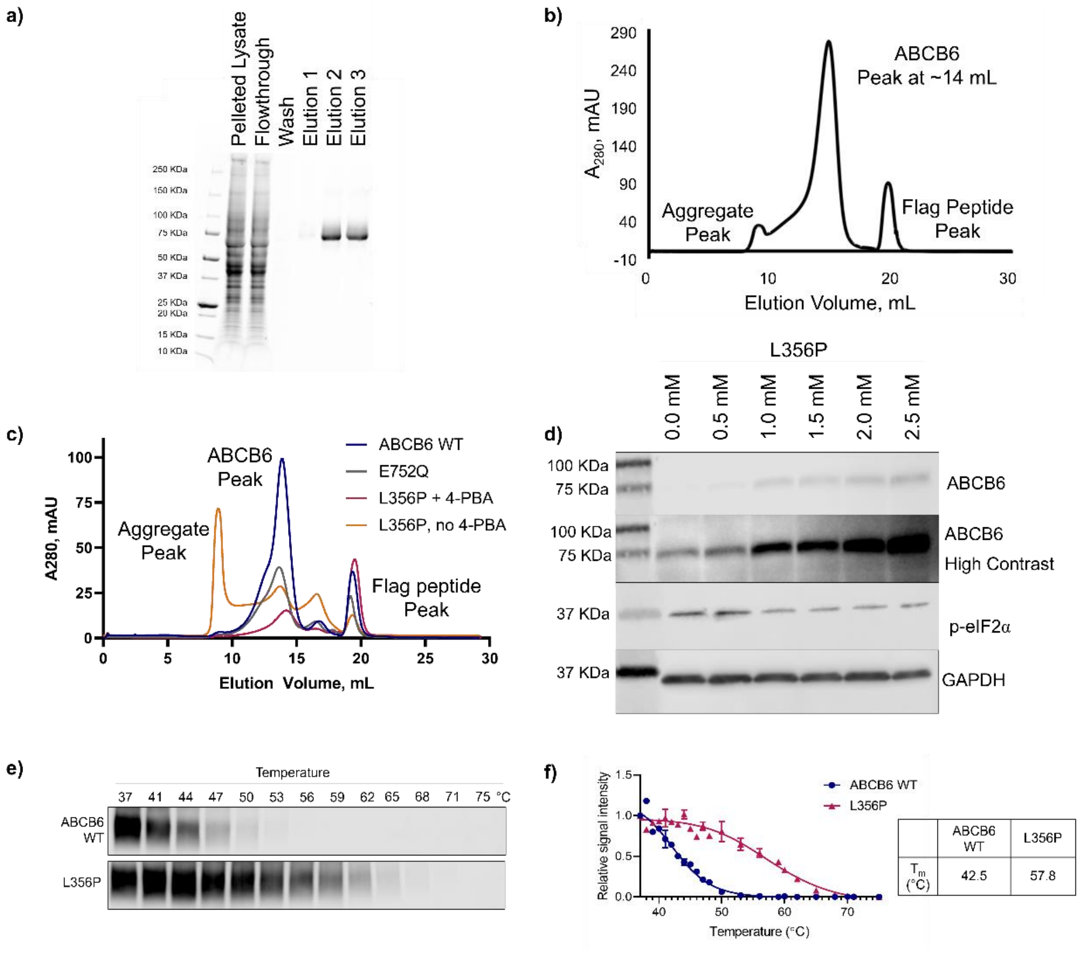


**Supplementary Figure 1.** Purification of ABCB6. **a)** SDS-PAGE of FLAG (M2) purification fractions of ABCB6 WT. **b)** Size exclusion chromatogram (SEC) of Flag-purified fractions from **(a)**. **c)** SEC chromatograms for ABCB6 WT, E752Q, and L356P with 4-PBA treatment all show a dominant peak at ~14 mL, while L356P expressed without 4-PBA contains mostly aggregated protein. **d)** Western blot shows L356P expression increases with 4-PBA treatment. When untreated, the L356P mutant evokes phosphorylation of eIF2α (p-eIF2α), a stress response to help restore protein homeostasis and proper folding. With the addition of 4-PBA, the p-eIF2α signal is attenuated. **e)** Thermal stability studies by western blot show L356P has increased thermal stability compared to ABCB6 WT. **f)** Protein signal quantification from thermal stability studies. The $T_m$ of L356P (57.8°C, N=2 technical replicates, magenta) is elevated 15.3°C compared to the $T_m$ of ABCB6 WT (42.5°C, N=3 technical replicates, indigo). Data are reported as mean ± SEM. Source data are provided in the Source Data file.

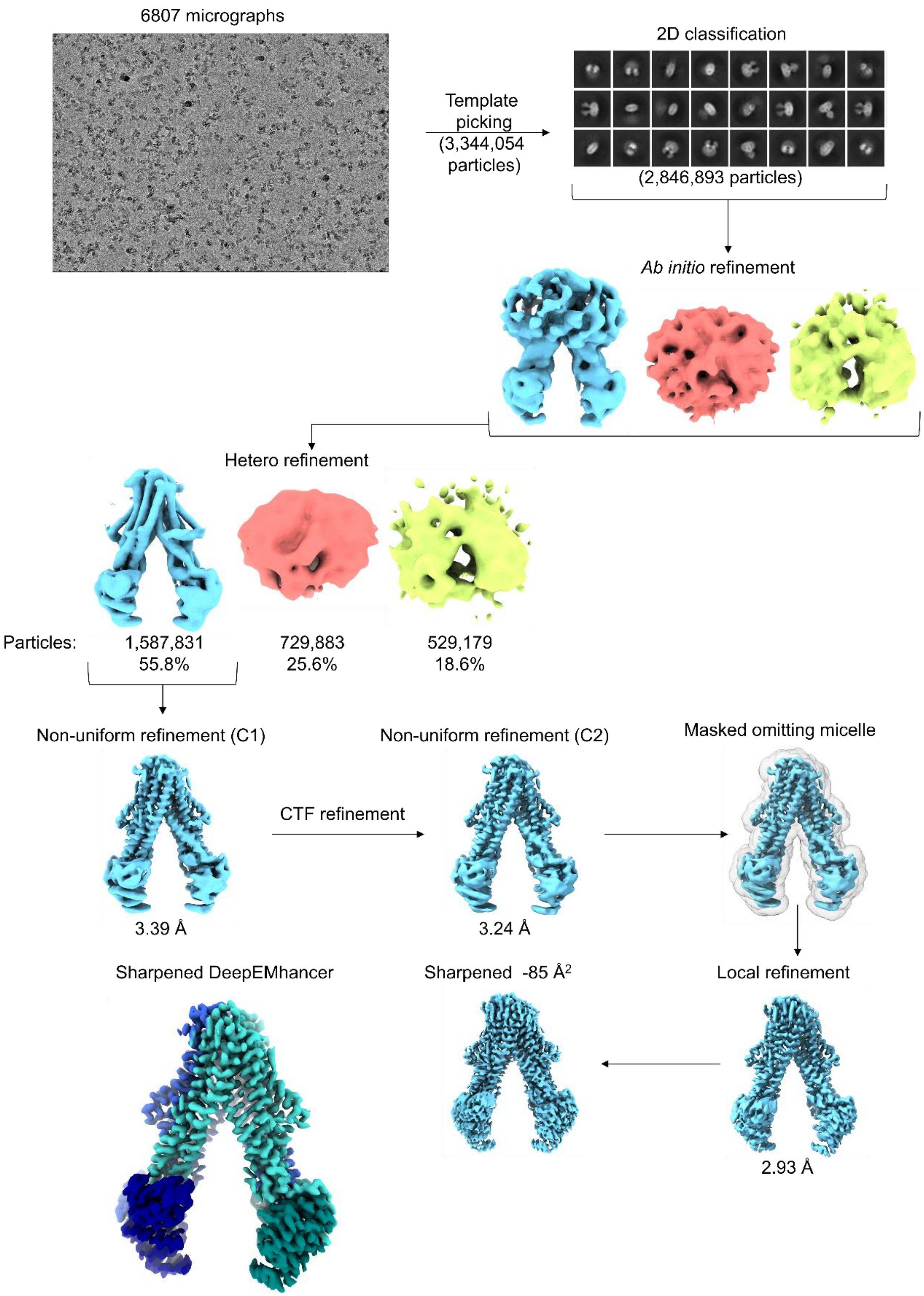


**Supplementary Figure 2.** Cryo-EM image processing. Processing workflow to obtain the cryo-EM density reconstruction. A total of 1,587,831 particles were used for the 2.93 Å reconstruction. The protein model is built in green sticks.

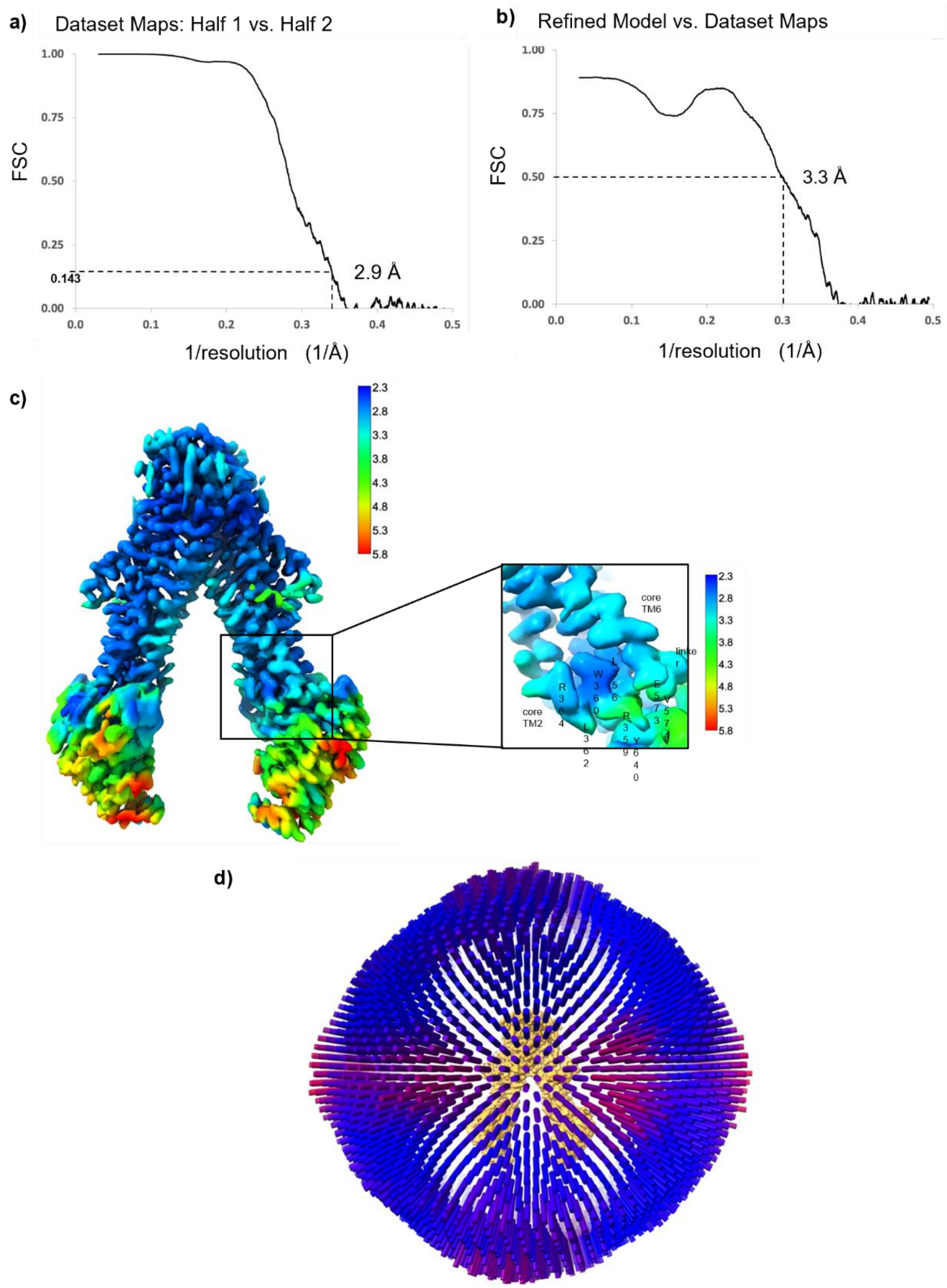


**Supplementary Figure 3.** Cryo-EM refinement. **a)** Fourier shell correlation (FSC) between the two half maps. **b)** FSC plot of the refined structure against the full map. **c)** Locally sharpened reconstruction colored by estimated local resolution (Blocres). Inset at right shows zoomed view of L356 and surrounding residues. **d)** Unsharpened reconstruction (gold) accompanied by a 3D representation of the particle angular distribution plot where the height and color (blue to red) of the cylinders is proportional to the number of particles in those views.

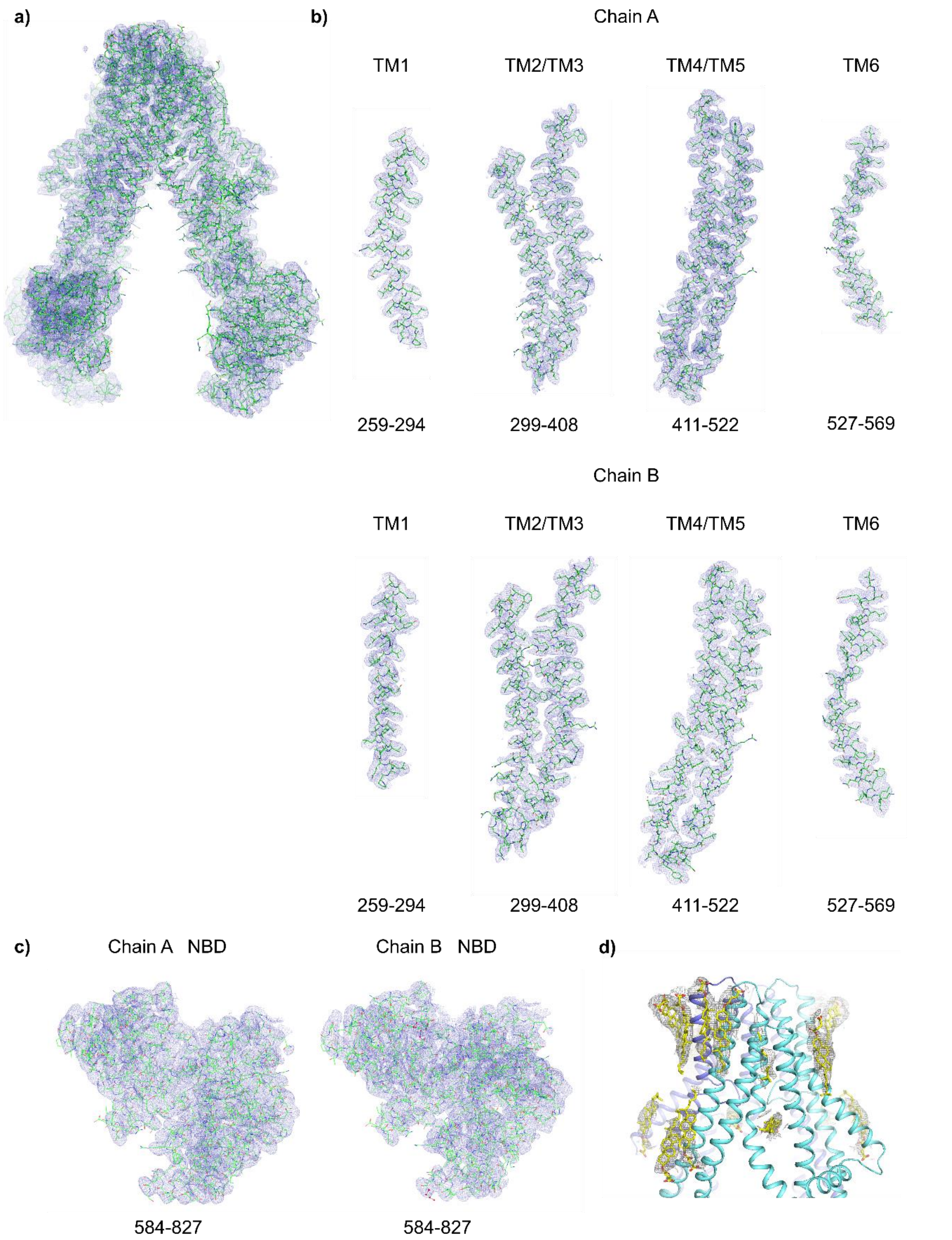


**Supplementary Figure 4.** Cryo-EM reconstruction. **a)** The locally sharpened reconstruction for the complex. The protein model is built in green sticks. **b)** Density for core ABC transporter TM helices. **c)** Density for both NBDs. **d)** Densities for twenty CHS molecules (yellow sticks) built in the outer and inner leaflet membrane regions of ABCB6 WT.

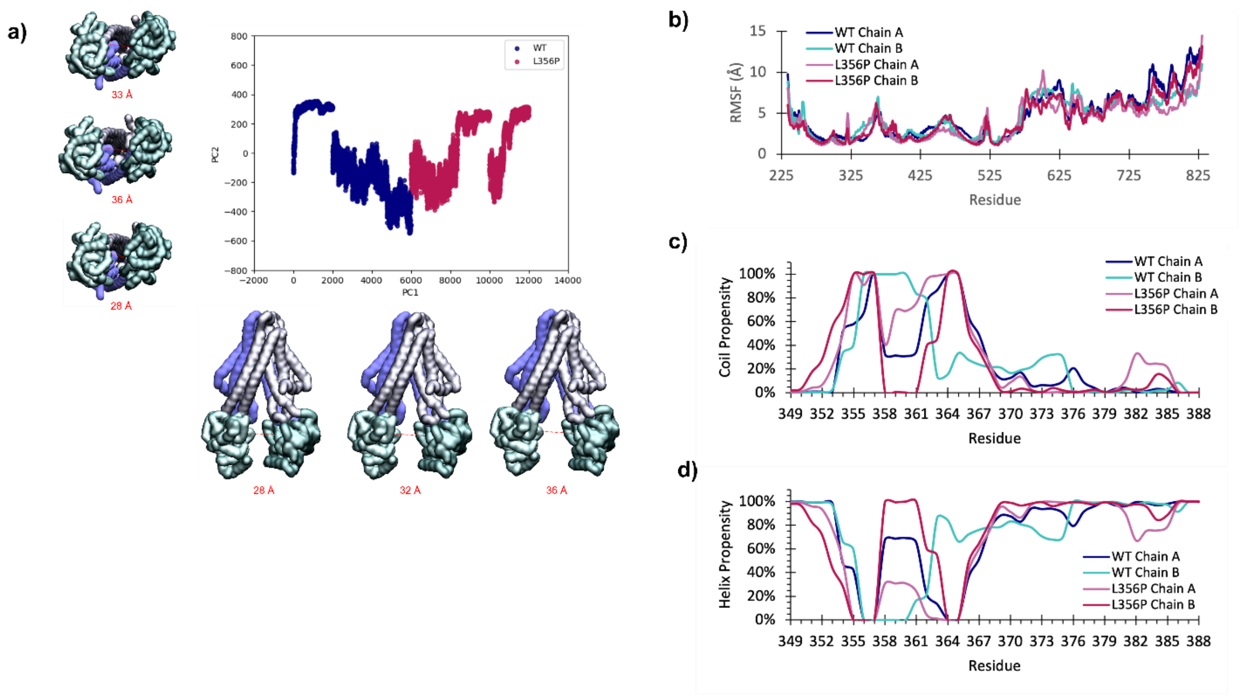


**Supplementary Figure 5**. Additional analysis of the L356P mutation. **a)** Principal component analysis for the ABCB6 WT and L356P mutant protein in the share subspace, where PC1 and PC2 represent the structural factors in which the most variation is observed over the course of the MD simulations. **b)** Calculated RMSF for ABCB6 WT (indigo/turquoise) and L356P (pink/magenta). Secondary structure propensity for the coupling helix region that contains L356 residue for the ABCB6 WT and L356P mutant protein. Coil propensity is shown in **(c),** and helix propensity is shown in **(d)**.

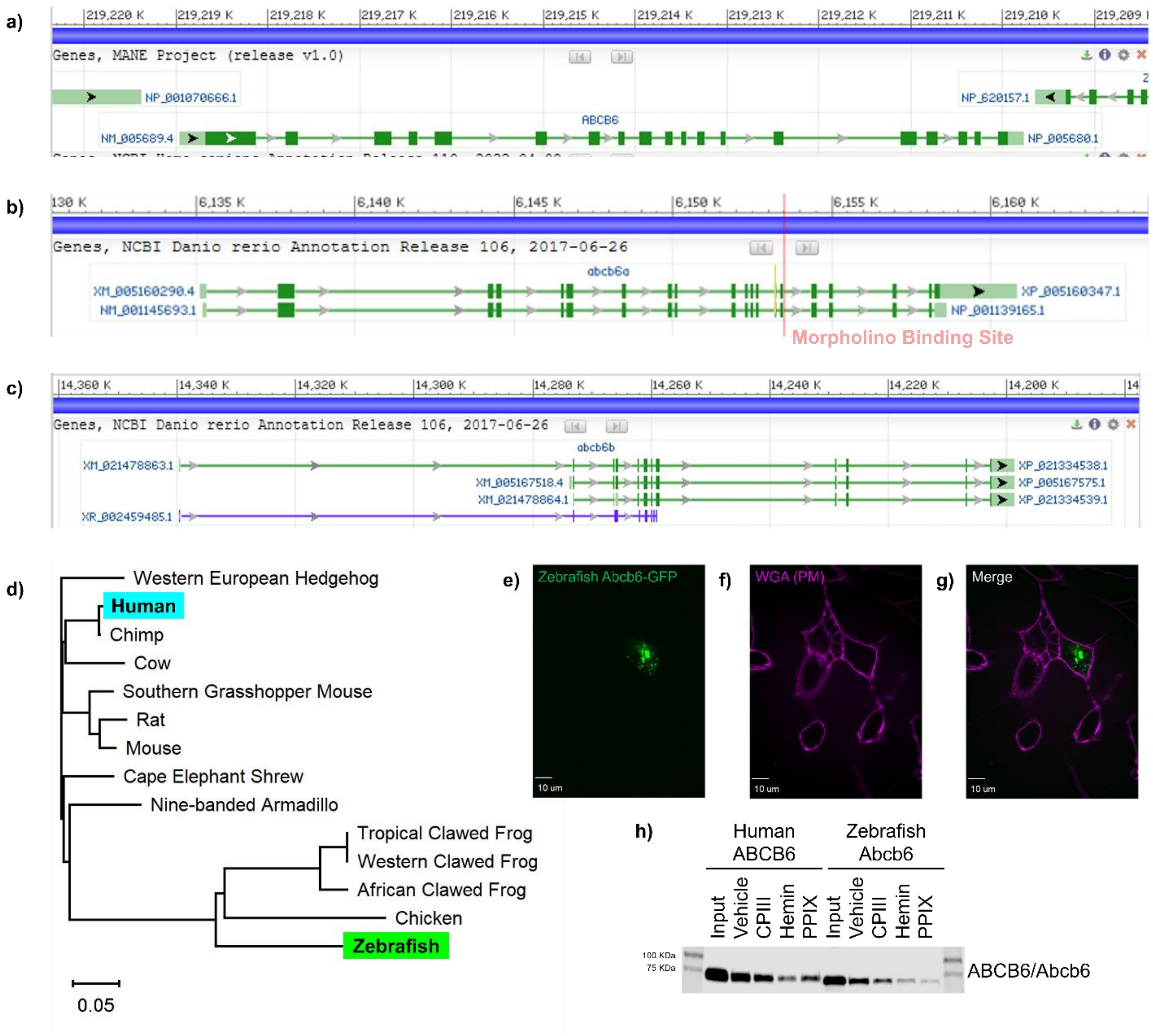


**Supplementary Figure 6.** Additional characterization of zebrafish Abcb6. Gene structures of human ABCB6 (Gene ID: 10058, **a)** is similar to zebrafish abcb6a (Gene ID: 564067, *(B)*, but not abcb6b (Gene ID: 100004801, **(c)**. Figures ***a-c*** generated using NCBI Sequence Viewer.[3] **d)** Cladogram of ABCB6 homologs. ABCB6 is highlighted in blue and zebrafish Abcb6 is highlighted in green. Live cell imaging **(e-g)** shows that zebrafish Abcb6-GFP does not traffic to the plasma membrane, but instead is located intracellularly. (**h)** Western blot of hemin-agarose pulldown with competition by various porphyrin compounds shows zebrafish Abcb6 binds porphyrins like its human counterpart. Western blot was developed using an Anti-ABCB6 antibody that recognized both human and zebrafish forms of the protein. Source data are provided in the Source Data file.

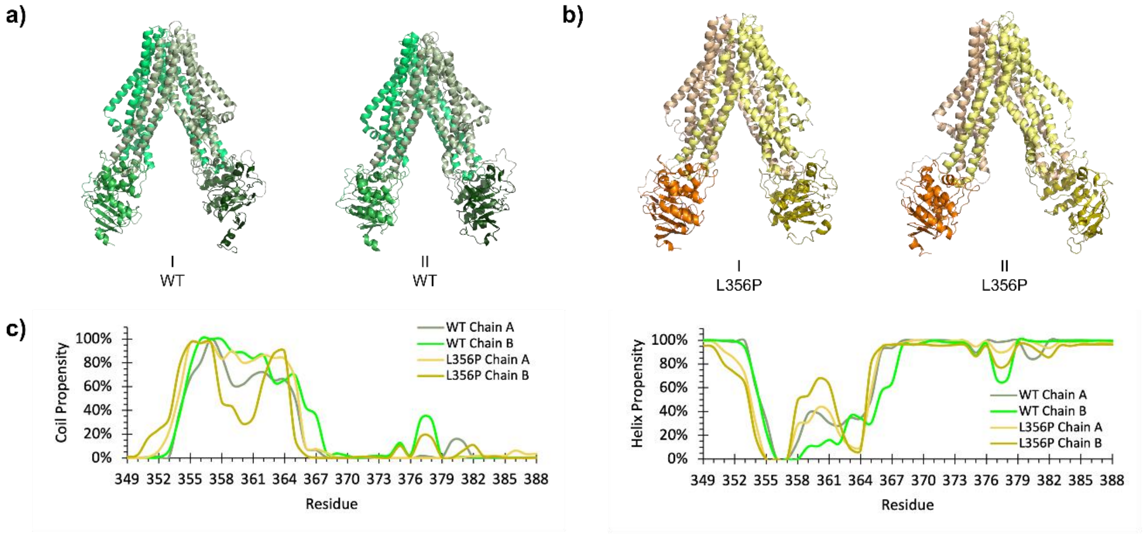


**Supplementary Figure 7**. Analysis of zebrafish Abcb6 homology model. **a)** Representative structure from the MD simulations on Zebrafish Abcb6. **b)** Representative structure from the MD simulations on Zebrafish Abcb6 L356P mutation. **c)** Secondary structure propensity as measured through DSSP for the coupling helix region that contains L356P for the zebrafish Abcb6 WT and L356P mutant protein.

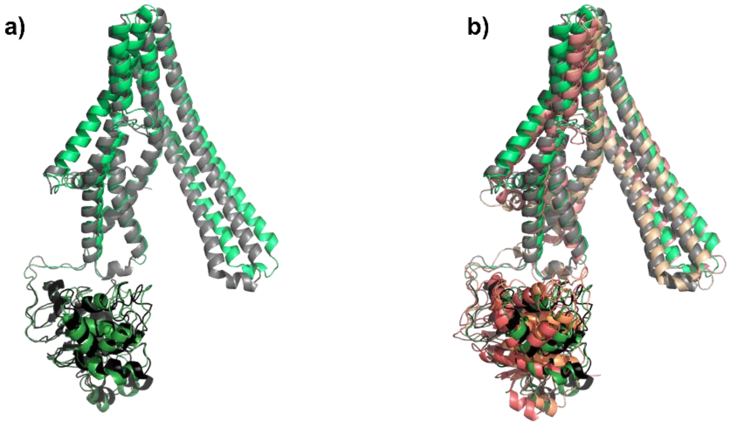


**Supplementary Figure 8.** Comparison of zebrafish homology model and AlphaFold model. **a)** Overlay of Abcb6 homology model (green) and AlphaFold structure (grey, accession code: F1QCK2) shows little difference between the two structures with the largest structural deviation observed in the orientation of the coupling helix in the TMD. The coupling helix samples both the homology model and AlphaFold orientation over the course of the 1500 ns of total MD simulation **(b)** as shown by two representative clusters from the simulation (beige and pink). Nucleotide binding domains (NBDs) are shown in darker tones while transmembrane domains (TMDs) are colored in lighter tones.

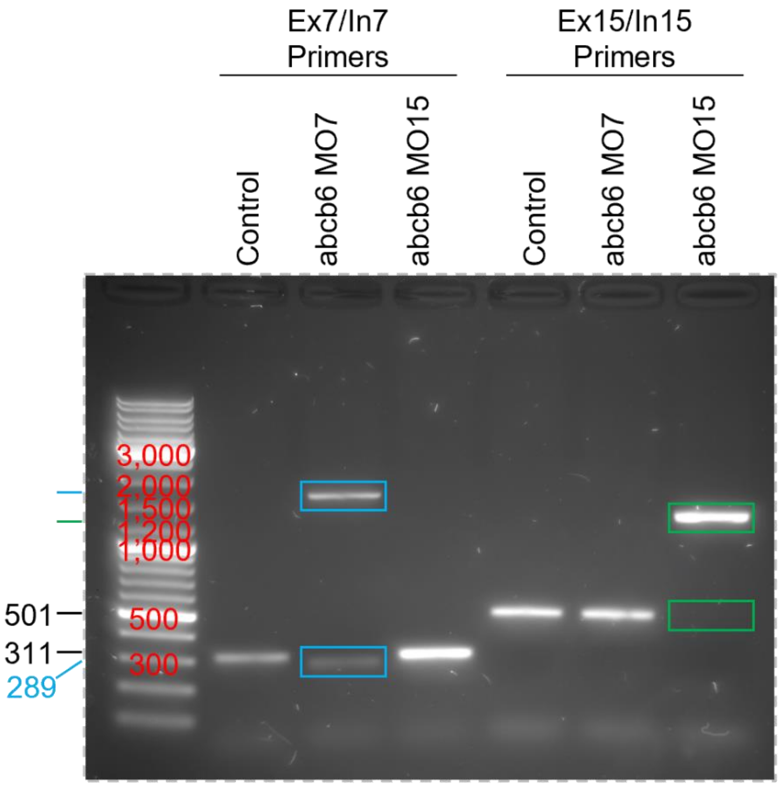


**Supplementary Figure 9.** RT-PCR analysis of morpholino oligonucleotides. AB (wildtype) zebrafish embryos were injected with splice site morpholinos targeted to either exon 7 or exon 15 of abcb6 (MO7 and MO15, respectively) or a control MO (GeneTools LLC). RT-PCR product of 3 dpf embryos show the effect of morpholino injection. Embryos injected with control MO show a single band at 311 bp for primers binding exon 7 and intron 7 (Ex7/In7). With MO7 treatment, two bands at 1643 bp and 281 bp (from cryptic GT in exon 7) are observed while the 311 bp band is not observed, suggesting MO7 correctly targets the splice junction of exon 7. For exon 15 and intron 15 primers (Ex15/In15), the RT-PCR product from the control morphant is a single band at 501 bp, whereas the MO15 morphant shows a 1359 bp band along with the disappearance of the 501 bp band (green boxes). These Ex15/In15 PCR products also suggest that MO15 correctly targets the splice junction of exon 15.

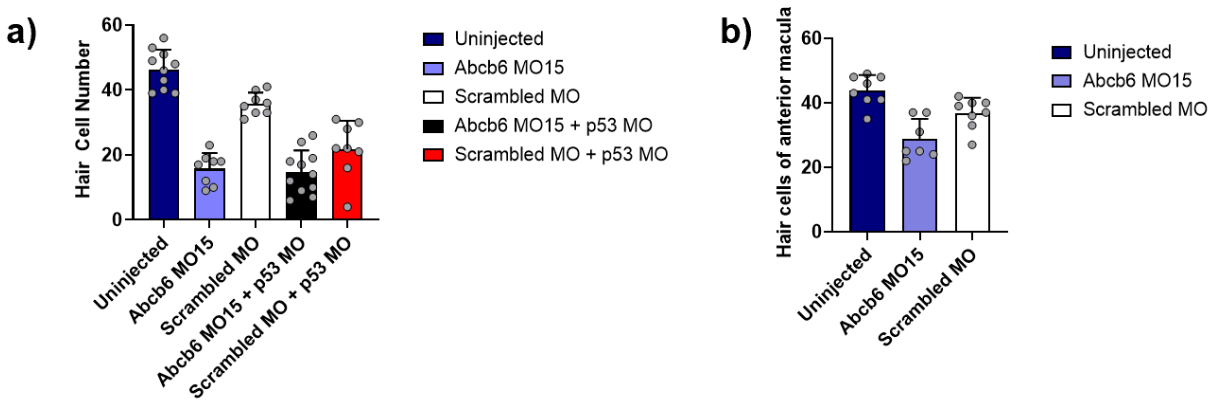


**Supplementary Figure 10.** Hair cell numbers in 3 dpf zebrafish morphant **(a)** lateral line and **(b)** inner ear. **a)** There is a significant effect of MO on lateral line hair cell number (one-way ANOVA, $F_{4,40}$=46.31, p<0.0001). No difference in lateral line hair cell number was observed between fish injected with the abcb6 morpholino MO15 alone and fish co-injected with the abcb6 and p53 MOs (1-tailed Student's t-test, p=0.35). For each fish, hair cells were counted in 7 neuromasts per fish, then counts were summed to arrive at one value per fish. The sums were used rather than average number of hair cells per neuromast to account for the fact that within a single fish there are many more hair cells in the head and more rostral trunk neuromasts than on the tail, particularly in 3-day-old animals. Hair cell counts are from 3 dpf brn3c transgenic larvae, N=10 fish for uninjected, N=8 fish for abcb6 MO15, N=8 fish for scrambled MO, N=11 fish for abcb6 + p53 MO, and N=8 fish for scrambled MO + p53 MO. **b)** Hair cell quantification from the anterior inner ear macula, which is associated with the utricular otolith. As with the lateral line, there is a significant effect of MO on inner ear hair cell number (one-way ANOVA, $F_{2,20}$=15.27, p<0.0001). Hair cell counts are from 3 dpf brn3c transgenic larvae, N=8 fish for uninjected and scrambled MO, N=7 fish for abcb6 MO15). Experiments were performed in Brn3c:mGFP transgenic embryos. For both graphs data reported as mean + SD. The person counting hair cells was blinded to treatment. Source data are provided in the Source Data file.

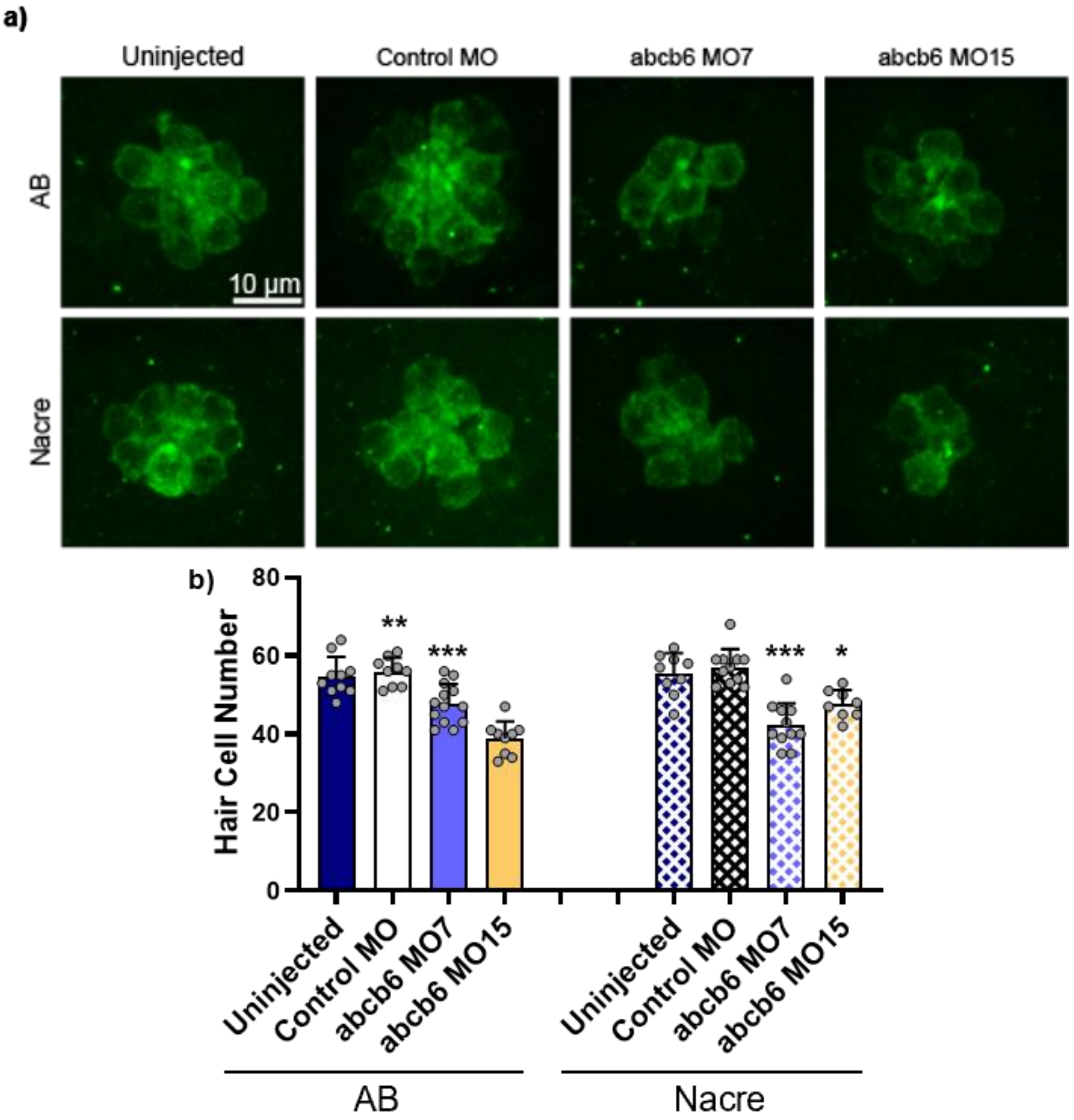


**Supplementary Figure 11.** Morpholino knockdown reduces lateral line hair cell number in 3 dpf zebrafish. AB (wildtype) or nacre (pigment mutant) zebrafish embryos were injected with splice site morpholinos targeted to either exon 7 or exon 15 of *abcb6* (MO7 and MO15, respectively). Controls received either a control MO or were left uninjected. **a)** Representative confocal images of the MI2 neuromast from each group; hair cells were labeled with an antibody to myosin7a. The scale bar in the top left image applies to all panels. **b)** Quantitation of hair cell number shows a significant effect of morpholino injection on hair cell number (one-way ANOVA, F=20.17, p<0.0001). Abcb6 morphants had fewer hair cells than uninjected controls (Bonferroni-adjusted posthoc tests, *p<0.05, **p<0.01, ***p<0.001). N=10 fish for AB Uninjected, N=9 fish for AB Control MO, N=13 fish for AB abcb6 MO, N=9 fish for abcb6 MO15, N=9 fish for Nacre Uninjected, N=11 fish for Nacre Control MO, N=11 fish for Nacre abcb6 MO7, N=8 fish for Nacre abcb6 MO15. Data are shown as mean +/- 1 SD. The person counting hair cells was blinded to treatment. Source data are provided in the Source Data file.

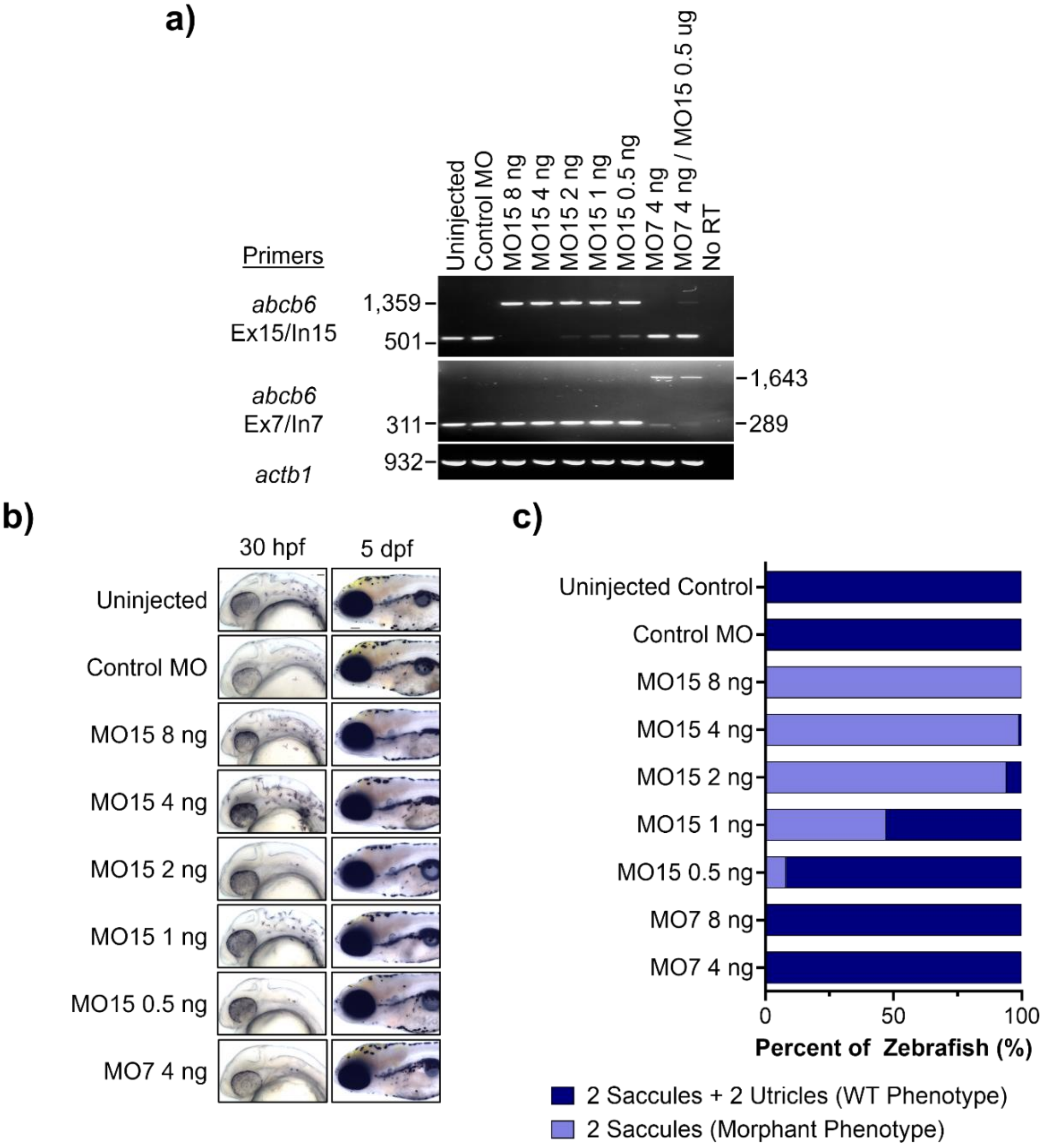


**Supplementary Figure 12.** 2-saccule phenotype is dependent on MO15 dose, but not MO7 dose. **a)** Effect of morpholino injection on RT-PCR products. RT-PCR product sizes are previously outlined in **Supplementary Figure 9**. Representative images **(b)** and quantitation of phenotypes **(c)** of zebrafish 30 hours post fertilization (hpf) or 5 dpf show the 2-saccule phenotype is dependent on injected MO15 dose. However, MO7 injection does not affect otolith development. 2-saccule phenotype was confirmed in AB, nacre, and TL zebrafish lines. Morpholino injections were repeated at least 3 times. Data for uninjected control (N=320 fish), control MO (N=132 fish), MO15 8 ng (N=74 fish), MO15 4 ng (N=191 fish), MO15 2 ng (N=177 fish), MO15 1 ng (N=127 fish), MO15 0.5 ng (N=113 fish), MO7 8 ng (N=66 fish), MO7 4 ng (N=131 fish), can be found in **Supplementary Table 7** or the Source Data file.

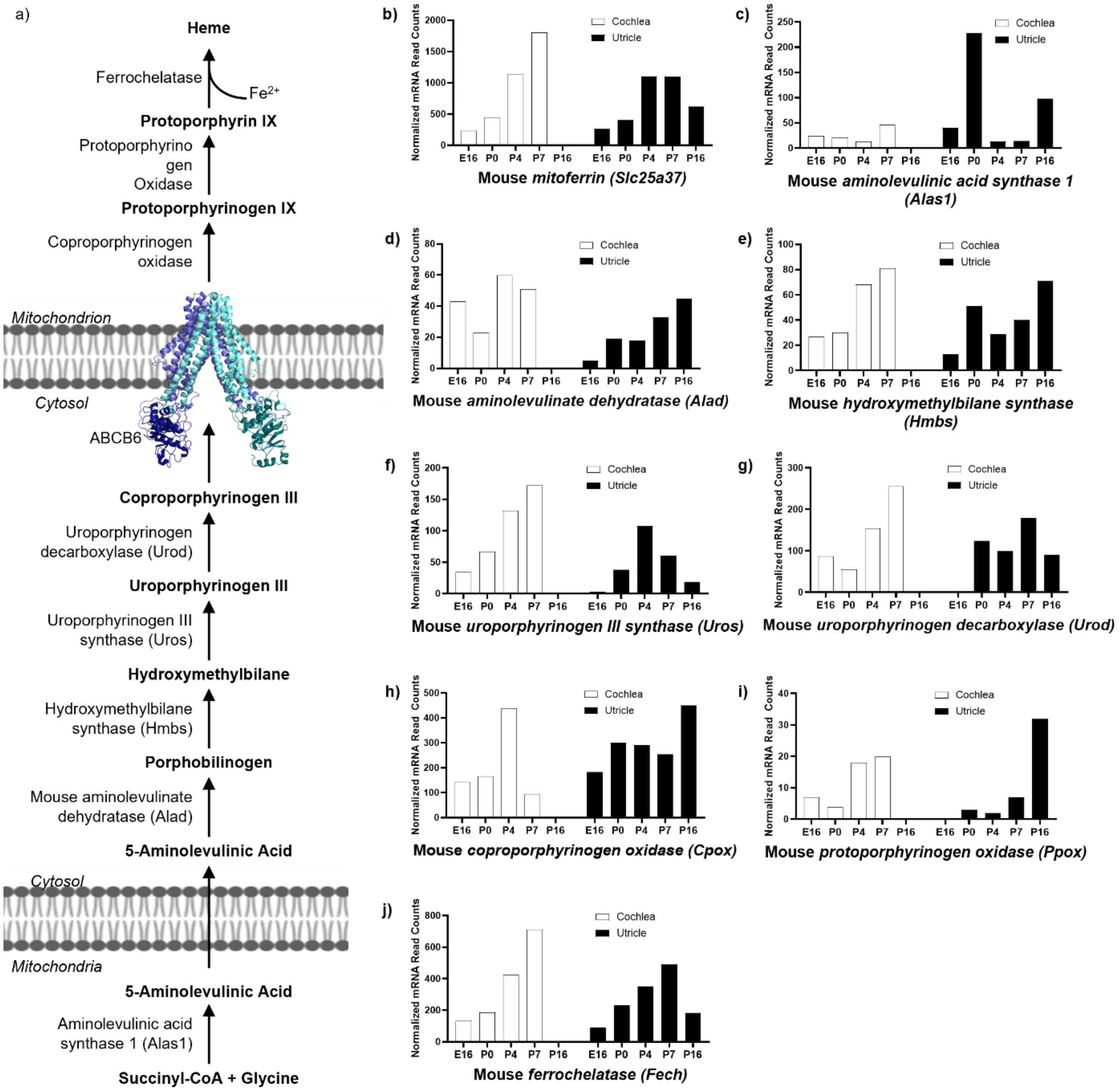


**Supplementary Figure 13**. Expression of heme synthesis genes increases with development in the cochlea. **a)** Schematic of the heme synthesis pathway. **b-j)** Normalized mRNA read counts from various genes in the heme biosynthesis pathway. Mitoferrin, a mitochondrial iron transporter that helps move the ferrous iron required for the final step in heme synthesis, was also included. Several genes, including mitoferrin **(a)**, hydroxymethylbilane synthase **(e)**, uroporphyrinogen III synthase **(f)**, and ferrochelatase **(j)** show increased read counts in the cochlea with development. Data taken from the SHIELD database.[11] Source data are provided in the Source Data file**.**

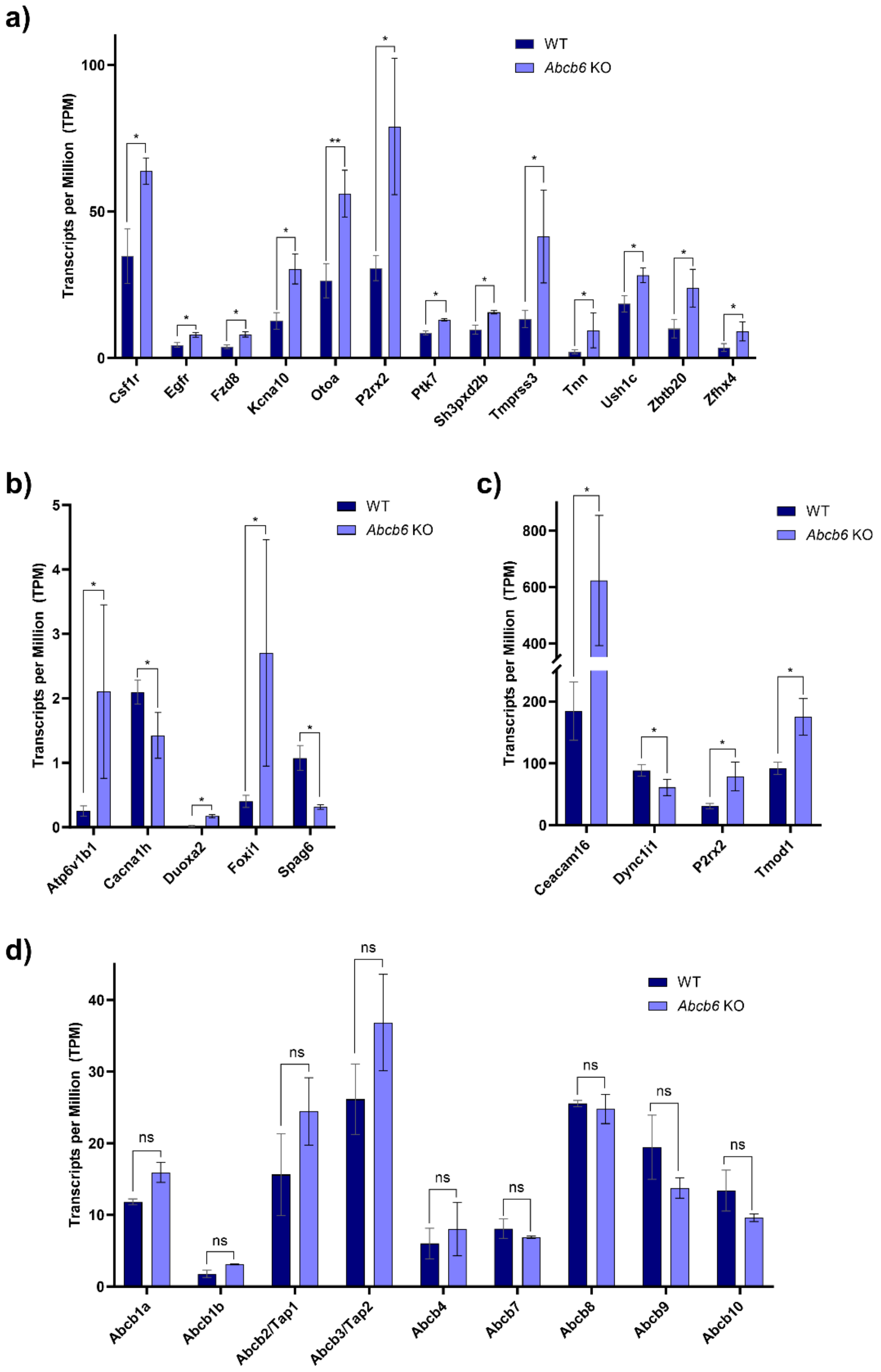


**Supplementary Figure 14.** RNA-seq shows several hearing-related genes are affected by knockout (KO) of Abcb6. Genes are grouped based on expression level, with genes in the 2-100 transcripts per million (TPM) range in **(a),** 0-5 TPM range in **(b),** and 30-900 TPM range in **(c)**. No significant changes were observed in the ABCB transporters of Abcb6 KO mice **(d)**. Source data are provided in the Source Data file.

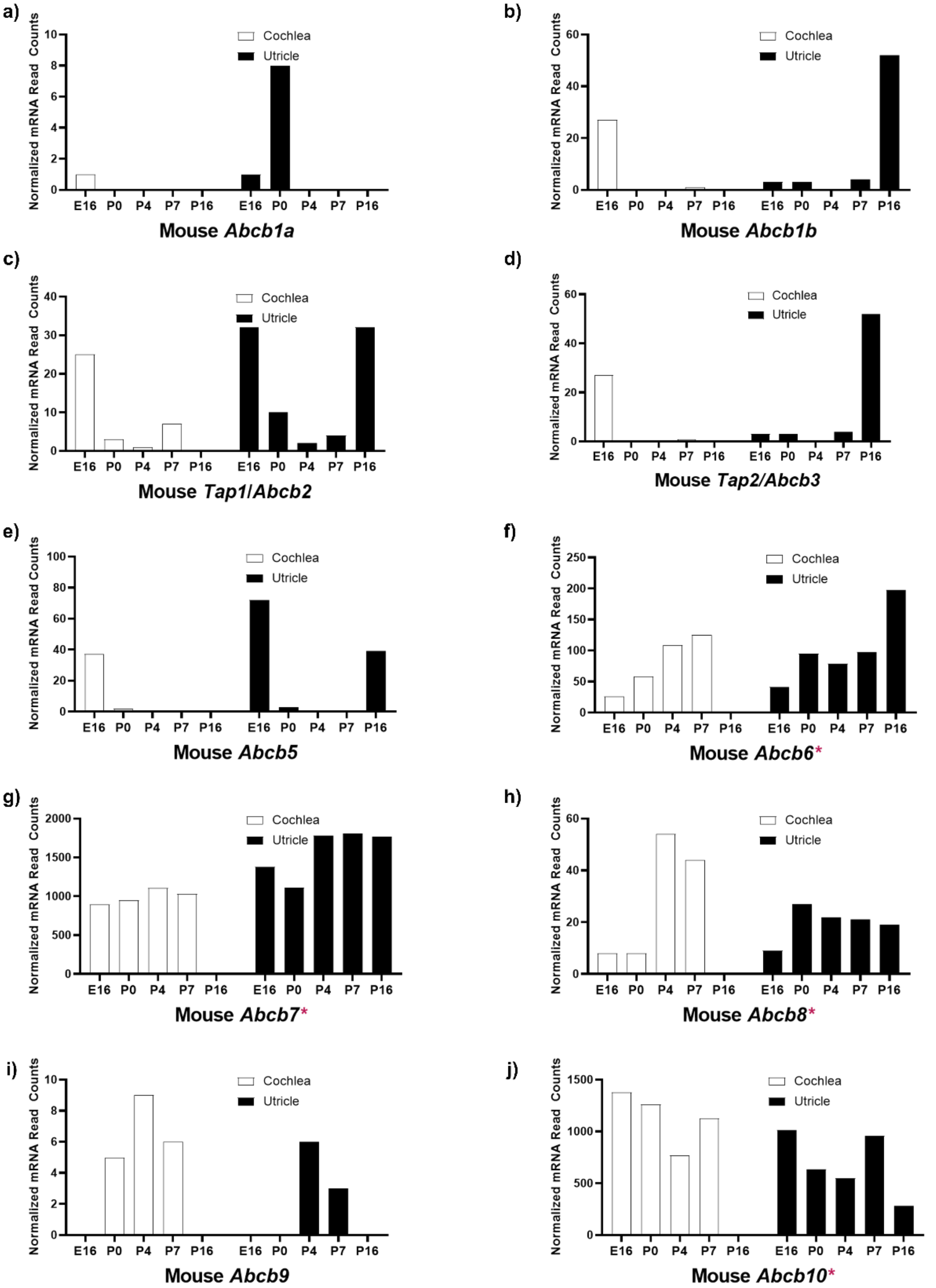


**Supplementary Figure 15.** Normalized mRNA read counts from various members of the ABC B-subfamily. The mitochondrial ABC transporters, denoted with a magenta *, Abcb6 **(f)**, Abcb7 **(g)**, Abcb8 **(h)**, Abcb10 **(j)** show elevated read counts compared to the other ABCB proteins (Abcb1a **(a)**, Abcb1b **(b)**, Tap1/Abcb2 **(c)**, Tap2/Abcb3 **(d)**, and Abcb5 **(e)**). However, only Abcb6 **(f)** shows an increase in read counts of the cochlea with development. Data taken from the SHIELD database.[11] Source data are provided in the Source Data file.

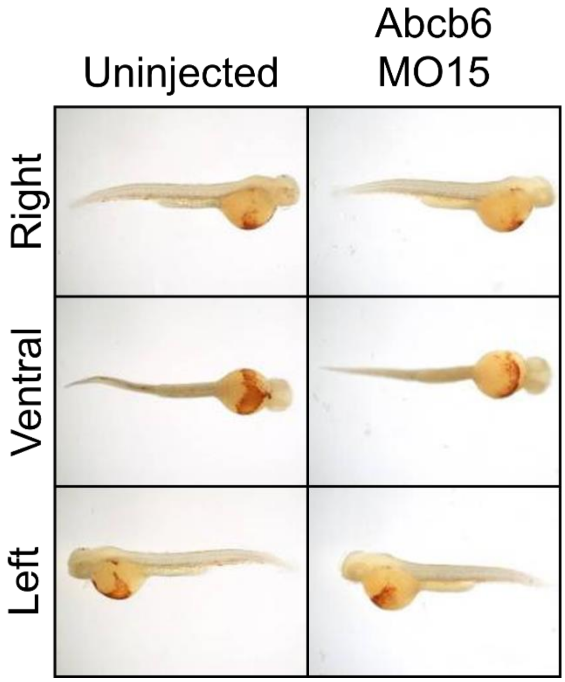


**Supplementary Figure 16.** *o*-Dianisidine staining does not reflect a change in red blood cells in 2 dpf abcb6 MO15 morphants (AB background). The heme co-factor of hemoglobin, contained in erythrocytes, reacts with *o*-dianisidine to produce an orange color.

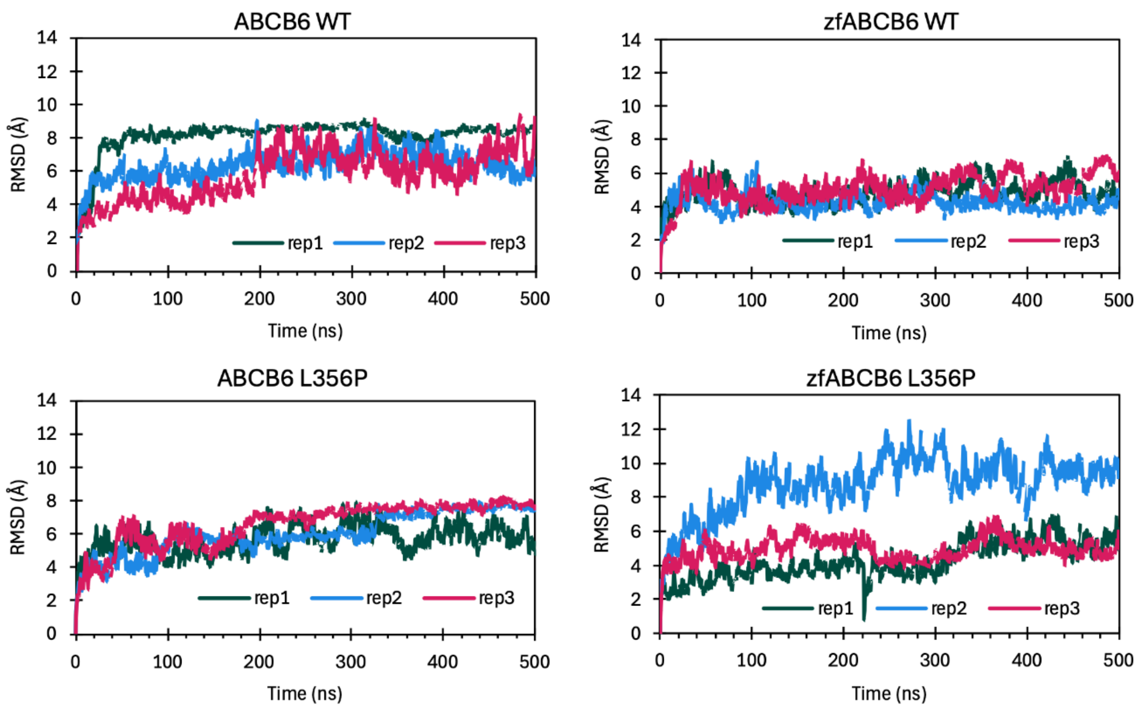


**Supplementary Fig. 17.** Time-dependent RMSD of human and zebrafish Abcb6 simulations. Time dependent backbone RMSD of human ABCB6 (left column) and zebrafish Abcb6 (right column) for the wild type ABCB6 (top row) and the L356P mutant (bottom row). In each case the RMSD plateaued within the first 100 ns of simulation. The magnitude of the RMSD arises from the separation of the NBDs, shown in **Fig. 2**, **Supplementary Fig. 5** and **Supplementary Fig. 7**. Cluster analysis and RMSF of human ABCB6 (**Supplementary Fig. 5**) and cluster analysis of zebrafish ABCB6 **(Supplementary Fig. 7**) confirm that these changes are related to the distance and relative orientation of the NBDs.

## Supplementary References


1. Fukuda, Y. *et al.* Conserved intramolecular disulfide bond is critical to trafficking and fate of ATP-binding cassette (ABC) transporters ABCB6 and sulfonylurea receptor 1 (SUR1)/ABCC8. *J Biol Chem* 286, 8481-8492, (2011).
2. Untergasser, A. *et al.* Primer3—new capabilities and interfaces. *Nucleic Acids Research* **40**, e115-e115, doi:10.1093/nar/gks596 (2012).
3. Hasson, T. *et al.* Unconventional Myosins in Inner-Ear Sensory Epithelia. *Journal of Cell Biology* 137, 1287-1307, doi:10.1083/jcb.137.6.1287 (1997).
4. Coffin, A. B., Dabdoub, A., Kelley, M. W. & Popper, A. N. Myosin VI and VIIa distribution among inner ear epithelia in diverse fishes. *Hearing Research* **224**, 15-26, doi:10.1016/j.heares.2006.11.004 (2007).
5. Kaur, T. *et al.* Fractalkine Signaling Regulates Macrophage Recruitment into the Cochlea and Promotes the Survival of Spiral Ganglion Neurons after Selective Hair Cell Lesion. *The Journal of Neuroscience* **35**, 15050-15061, doi:10.1523/jneurosci.2325-15.2015 (2015).
6. Paffett-Lugassy, N. N. & Zon, L. I. Analysis of Hematopoietic Development in the Zebrafish. In *Developmental Hematopoiesis: Methods and Protocols* (ed Margaret H. Baron) 171-198 (Humana Press, 2005).
7. Adzhubei, I. A. *et al.* A method and server for predicting damaging missense mutations. *Nat Methods* 7, 248-249, doi:10.1038/nmeth0410-248 (2010).
8. Ng, P. C. & Henikoff, S. Predicting deleterious amino acid substitutions. *Genome Res* 11, 863-874, doi:10.1101/gr.176601 (2001).
9. Rangwala, S. H. *et al.* Accessing NCBI data using the NCBI Sequence Viewer and Genome Data Viewer (GDV). *Genome Res* 31, 159-169, doi:10.1101/gr.266932.120 (2021).
10. Reva, B., Antipin, Y. & Sander, C. Predicting the functional impact of protein mutations: application to cancer genomics. *Nucleic Acids Res* **39**, e118 (2011).
11. Shen, J., Scheffer, D. I., Kwan, K. Y. & Corey, D. P. SHIELD: an integrative gene expression database for inner ear research. *Database (Oxford)* 2015, bav071, doi:10.1093/database/bav071 (2015).
