# Supplementary material for: The role of ATP-binding Cassette subfamily B member 6 in the inner ear
Source: Nat Commun. 2024 Nov 18;15:9885. doi: 10.1038/s41467-024-53663-x (PMC11574312; doi:10.1038/s41467-024-53663-x)
Supplement: Supplementary file 3 — Description of additional supplementary files [file 41467_2024_53663_MOESM3_ESM.pdf]

Descripton of Additional Supplementary Files

Supplementary Movie 1: Swimming patterns of uninjected zebrafish

Supplementary Movie 2: Swimming patterns of Abcb6 MO15 zebrafish
