# Supplementary material for: The role of ATP-binding Cassette subfamily B member 6 in the inner ear
Source: Nat Commun. 2024 Nov 18;15:9885. doi: 10.1038/s41467-024-53663-x (PMC11574312; doi:10.1038/s41467-024-53663-x)
Supplement: Supplementary file 6 — Reporting summary [file 41467_2024_53663_MOESM6_ESM.pdf]

nature portfolio

Corresponding author(s): John Schuetz

Last updated by author(s): Sep 24, 2024

Reporting Summary

Nature Portfolio wishes to improve the reproducibility of the work that we publish. This form provides structure for consistency and transparency in reporting. For further information on Nature Portfolio policies, see our Editorial Policies and the Editorial Policy Checklist.

Statistics

For all statistical analyses, confirm that the following items are present in the figure legend, table legend, main text, or Methods section.

n/a	Confirmed	
☐	☒	The exact sample size (*n*) for each experimental group/condition, given as a discrete number and unit of measurement
☐	☒	A statement on whether measurements were taken from distinct samples or whether the same sample was measured repeatedly
☐	☒	The statistical test(s) used AND whether they are one- or two-sided<br>*Only common tests should be described solely by name; describe more complex techniques in the Methods section.*
☒	☐	A description of all covariates tested
☒	☐	A description of any assumptions or corrections, such as tests of normality and adjustment for multiple comparisons
☐	☒	A full description of the statistical parameters including central tendency (e.g. means) or other basic estimates (e.g. regression coefficient) AND variation (e.g. standard deviation) or associated estimates of uncertainty (e.g. confidence intervals)
☐	☒	For null hypothesis testing, the test statistic (e.g. F, t, r) with confidence intervals, effect sizes, degrees of freedom and P value noted<br>*Give P values as exact values whenever suitable.*
☒	☐	For Bayesian analysis, information on the choice of priors and Markov chain Monte Carlo settings
☒	☐	For hierarchical and complex designs, identification of the appropriate level for tests and full reporting of outcomes
☒	☐	Estimates of effect sizes (e.g. Cohen's *d*, Pearson's *r*), indicating how they were calculated

Our web collection on statistics for biologists contains articles on many of the points above.

Software and code

Policy information about availability of computer code

Data collection	Western blot signals were collected using Image Studio Software V5.2.5<br>Cryo-EM grids were screened on a Titan Krios microscope (FEI) using a Gatan K2 Summit Detector equipped with a GIF quantum energy filter. Images were automatically recorded using SerialEM.<br>Stained larvae images were obtained using a Nikon SMZ1500 stereomicroscope equipped with a Nikon DS-Fi2 color camera and Nikon NIS-Elements software.<br>Neuromast images were obtained using a Leica SP8 confocal microscope<br>Auditory brainstem responses were fed into a low-impedance Medusa digital biological amplifier system (RA4L; DTD).<br><br>Additional information is included in the materials and methods section as well as supplementary methods.
Data analysis	Western Blots: Image Studio Software V5.2.5, GraphPad Prism 8<br>Cryo-EM and model building: CryoSPARC, SerialEM, Phenix, DeepEMhancer, Molprobity, Chimera, PyMOL<br>Molecular dynamics simulations: AmberTools20, Python 3, Visual Molecular Dynamics (VMD) program, PyMOL<br>Homology modeling" UCSF Modeller 9.23<br>Stained larvae images: Nikon NIS-Elements software<br>Neuromast data: GraphPad Prism V9<br>Zebrafish behavioral analysis: MTrack plugin (V1.5.1) for ImageJ, GraphPad Prism V8<br><br>Additional information is included in the materials and methods section as well as supplementary methods.

For manuscripts utilizing custom algorithms or software that are central to the research but not yet described in published literature, software must be made available to editors and reviewers. We strongly encourage code deposition in a community repository (e.g. GitHub). See the Nature Portfolio guidelines for submitting code & software for further information.

Data

Policy information about availability of data

All manuscripts must include a data availability statement. This statement should provide the following information, where applicable:

- Accession codes, unique identifiers, or web links for publicly available datasets
- A description of any restrictions on data availability
- For clinical datasets or third party data, please ensure that the statement adheres to our policy

The cryoEM structure is available in the Electron Microscopy Data Bank (EMDB ID: EMD-46724) and the Protein Data Bank (PDB ID: 9DBQ). Simulation data is available at https://github.com/OMaraLab/ABCB6 (DOI: 10.5281/zenodo.13363754).

Research involving human participants, their data, or biological material

Policy information about studies with human participants or human data. See also policy information about sex, gender (identity/presentation), and sexual orientation and race, ethnicity and racism.

Reporting on sex and gender	Not Applicable
Reporting on race, ethnicity, or other socially relevant groupings	Not Applicable
Population characteristics	Not Applicable
Recruitment	Not Applicable
Ethics oversight	Not Applicable

Note that full information on the approval of the study protocol must also be provided in the manuscript.

Field-specific reporting

Please select the one below that is the best fit for your research. If you are not sure, read the appropriate sections before making your selection.

☒ Life sciences ☐ Behavioural & social sciences ☐ Ecological, evolutionary & environmental sciences

For a reference copy of the document with all sections, see nature.com/documents/nr-reporting-summary-flat.pdf

Life sciences study design

All studies must disclose on these points even when the disclosure is negative.

Sample size	Sample sizes were chosen based on standards in the field, with N=5 or 6 for mice and N>8 for zebrafish studies.
Data exclusions	The only data excluded from the study were two datapoints from the ATPase dataset identified by GraphPad Prism using a ROUT method with Q=0.1% to remove only definite outliers. Zebrafish embryos damaged during microinjection were removed from further analysis.
Replication	All experiments were repeated at least once and were successful.<br><br>Small-scale expression of DUH mutants: N=5 biological replicates, with each experiment repeated from transfection to western blot.<br>ATP- and hemin-agarose pulldowns of DUH mutants: N=3 biological replicates (ATP-Agarose) or N=4 biological replicates (Hemin-agarose), with each experiment repeated from transfection to western blot.<br>ATP- and hemin-agarose pulldowns of zebrafish Abcb6: N=3 biological replicates, with each experiment repeated from transfection to western blot.<br>L356P and ABCB6 WT treatments: N=3 biological replicates, with each experiment repeated from transfection to western blot.<br>ATPase assays: N=15 experiments for ABCB6 WT from five biological replicates, N=8 experiments for E752Q from three biological replicates, and N=4 for L356P from two biological replicates (two technical replicates per biological replicate).<br>Thermal stability studies: Two technical replicates of L356P and three technical replicates of ABCB6 WT, using the same batches of protein as used for ATPase and thermal shift assays.<br>Thermal shift assays: Three technical replicates (per protein) on the same batch of purified protein as used for the ATPase assay and thermal stability studies.<br>Peptide-N-glycosidase F (PNGase F) Assay was not repeated as it was a qualitative confirmation of the zebrafish Abcb6 sequence that showed the "NXC" glycosylation site was not present.<br>ATP- and hemin-agarose pulldowns of zebrafish Abcb6: N=3 biological replicates, with each experiment repeated from transfection to western blot.<br>Zebrafish studies: each experiment was repeated, and the results showed the same pattern. Exact cell numbers vary from animal to animal, even within a group.

	N values are included in the captions for the respective figures.
Randomization	Fertilized eggs were randomly assigned to the control or MO group. Eggs were harvested every 15-25 minutes in the morning during spawning. 10-20 eggs were injected with morpholino (MO), then the next 10-20 eggs would remain uninjected (or injected with scrambled MO), then the next set was injected with abcb6 MO, and so on.
Blinding	The person counting hair cells was blinded to treatment

Reporting for specific materials, systems and methods

We require information from authors about some types of materials, experimental systems and methods used in many studies. Here, indicate whether each material, system or method listed is relevant to your study. If you are not sure if a list item applies to your research, read the appropriate section before selecting a response.

Materials & experimental systems

n/a	Involved in the study
☐	☒ Antibodies
☐	☒ Eukaryotic cell lines
☒	☐ Palaeontology and archaeology
☐	☒ Animals and other organisms
☒	☐ Clinical data
☒	☐ Dual use research of concern
☒	☐ Plants

Methods

n/a	Involved in the study
☒	☐ ChIP-seq
☒	☐ Flow cytometry
☒	☐ MRI-based neuroimaging

Antibodies

Antibodies used	Antibodies were purchased from Rockland Immunochemicals (Anti-ABCB6, 600-401-945), Proteintech (Anti-GAPDH, 60004-1-Ig), Jackson ImmunoResearch Laboratories Inc (Peroxidase AffiniPure Donkey Anti-Mouse IgG (H+L), 715-035-150; Peroxidase AffiniPure Donkey Anti-Rabbit IgG (H+L), 715-035-152), Cell Signaling Technology (Anti-Phospho-eIF2α (Ser51) Antibody #9721), Santa Cruz Biotechnology (GFP Antibody (B-2) HRP, sc-9996 HRP),and Genescript (custom-developed Anti-ABCB6 Rabbit antibody). For zebrafish, the myosin7a antibody (25-6790) was purchased from Proteus Bioscience and anti-GFP (rabbit IgI fraction, 2339829) was purchased from ThermoFisher; secondary antibody (Alexa Fluor 488 Goat Anti-Rabbit IgG (H+L), A11008) was purchased from ThermoFisher.
Validation	Antibodies were used as the manufacturer's recommendation or from previous publications. The custom-developed Anti-ABCB6 was validated by western blot using cells transiently transfected with flag-tagged human ABCB6 or zebrafish Abcb6 pcDNA-3.1 expression vectors using the Anti-flag or Rockland Anti-ABCB6 as a control.

Eukaryotic cell lines

Policy information about cell lines and Sex and Gender in Research

Cell line source(s)	Expi293F cells were purchased from ThermoFisher Scientific (Cat. no. A14527). HEK cells and NIH 3T3 cells were received from collaborators 25 years ago.
Authentication	Cell lines were not authenticated but were validated to have no detectable basal ABCB6 expression by western blot developed with custom-developed Anti-ABCB6 antibody.
Mycoplasma contamination	HEK-293 and NIH 3T3 cells tested negative for mycoplasma contamination. Expi293 cells were not tested.
Commonly misidentified lines (See ICLAC register)	No cell lines currently identified in ICLAC register were used in this study.

Animals and other research organisms

Policy information about studies involving animals; ARRIVE guidelines recommended for reporting animal research, and Sex and Gender in Research

Laboratory animals	Mouse (Mus musculus): 5–6-week ABCB6 KO (as previously reported) or C57/129 mix control mice were used. Zebrafish (Danio rerio) AB background, Nacre, p53 (-/-), and Tg(Brn3c:GFP) were used for zebrafish studies. Relevant figure captions state the age and strain for any animals used for the given figure.
Wild animals	No wild animals were used in this study
Reporting on sex	Mouse (Mus musculus): ABCB6 KO mice included female (n=3) and male (n=3) mice. WT C57/129 control mice included female (N=3) and male (N=2) mice. (Danio rerio): In lab zebrafish, sex determination is partially based on environment and sex cannot be

	detected in animals this young. However, based on research in the Coffin lab we generate approximately equal sex ratios once the fish are older.
Field-collected samples	No field-collected samples were used in this study.
Ethics oversight	Mouse: All procedures for this study (animal protocol #297) were reviewed and approved by the Institutional Animal Care and Use Committee (IACUC) of St. Jude Children's Research Hospital. Zebrafish: All experiments were performed in accordance with the University of Wisconsin-Madison Institutional Animal Care and Use Committee (IACUC) (protocol #M005020), Washington State University IACUC (protocol #6024), or by the Animal Care and Use Committee at the National Institutes of Health (NIH; protocol #1362-13).

Note that full information on the approval of the study protocol must also be provided in the manuscript.

Plants

Seed stocks	Not applicable
Novel plant genotypes	Not applicable
Authentication	Not applicable
